# General Aspects of Colorectal Cancer

**DOI:** 10.5402/2012/139268

**Published:** 2012-11-14

**Authors:** Josep J. Centelles

**Affiliations:** Departament de Bioquímica i Biologia Molecular, Facultat de Biologia, Universitat de Barcelona, Avenida Diagonal 643, Catalunya, 08028 Barcelona, Spain

## Abstract

Colorectal cancer (CRC) is one of the main causes of death. Cancer is initiated by several DNA damages, affecting proto-oncogenes, tumour suppressor genes, and DNA repairing genes. The molecular origins of CRC are chromosome instability (CIN), microsatellite instability (MSI), and CpG island methylator phenotype (CIMP). A brief description of types of CRC cancer is presented, including sporadic CRC, hereditary nonpolyposis colorectal cancer (HNPCC) or Lynch syndromes, familiar adenomatous polyposis (FAP), MYH-associated polyposis (MAP), Peutz-Jeghers syndrome (PJS), and juvenile polyposis syndrome (JPS). Some signalling systems for CRC are also described, including Wnt-**β**-catenin pathway, tyrosine kinase receptors pathway, TGF-**β** pathway, and Hedgehog pathway. Finally, this paper describes also some CRC treatments.

## 1. An Introduction on Colorectal Cancer

Colorectal cancer (CRC) is one of the leading causes of cancer lethality. In the United States, 143,460 new cases of CRC are foreseen to be diagnosed during 2012 (73,420 men and 70,040 women), and 51,690 patients will die of this disease. From 2005 to 2009, the median age at death for CRC was 74 years of age (approximately 0.0% died under age 20; 0.6% between 20 and 34; 2.5% between 35 and 44; 8.6% between 45 and 54; 16.5% between 55 and 64; 22.0% between 65 and 74; 29.0% between 75 and 84; and 20.8% from 85 years of age and older [1]).

CRC can be separated into 72% for the colon cancer and 28% for the rectum cancer, although incidence of CRC is generally reported together. Classification of CRC is referred to their pathological stage, which can be observed after surgery [2]. The clinical and the pathological stages may be different, as the imaging tests can be different from the observed stage after surgery.

The most common used staging system for CRC is that of the American Joint Committee on Cancer (AJCC), known also as the TNM system. Nevertheless, other staging systems, such as the Dukes [3] and Astler-Coller [4] systems, are still in use. These old systems are not as precise as the TNM system [5, 6] (see Table 1 for correspondences between the three staging systems).

The three letters combined in AJCC system mean the following:

T describes how far the main (primary) tumour has grown into the wall of the intestine and whether it has grown into nearby areas;
N describes the extent of spread to nearby (regional) lymph nodes. Lymph nodes are small bean-shaped collections of immune system cells that are important in fighting infections. To get an accurate idea about lymph node involvement, it is recommended to look under a microscope at least 12 lymph nodes (removed during surgery);
M indicates whether the cancer has spread (metastasized) to other organs of the body (CRC can spread almost anywhere in the body, but the most common sites of spread are the liver and lungs).

These three letters are combined with numbers (from 0 to 4) indicating increasing severity, whereas a letter “X” (instead of a number) means that the information is not available.

Tx: No description of the tumor's extent is possible because of incomplete information.
Tis: The cancer is in the earliest stage (*in situ*). It involves only the mucosa. It has not grown beyond the muscularis mucosa (inner muscle layer).
T1: The cancer has grown through the muscularis mucosa and extends into the submucosa.
T2: The cancer has grown through the submucosa and extends into the muscularis propria (thick outer muscle layer).
T3: The cancer has grown through the muscularis propria and into the outermost layers of the colon or rectum but not through them. It has not reached any nearby organs or tissues.
T4a: The cancer has grown through the serosa (also known as the visceral peritoneum), the outermost lining of the intestines.
T4b: The cancer has grown through the wall of the colon or rectum and is attached to or invades into nearby tissues or organs.
Nx: No description of lymph node involvement is possible because of incomplete information.
N0: No cancer in nearby lymph nodes.
N1: Cancer cells are found in or near 1 to 3 nearby lymph nodes.
   N1a: Cancer cells are found in 1 nearby lymph node.
   N1b: Cancer cells are found in 2 to 3 nearby lymph nodes.
   N1c: Small deposits of cancer cells are found in areas of fat near lymph nodes, but not in the lymph nodes themselves.
N2: Cancer cells are found in 4 or more nearby lymph nodes.
   N2a: Cancer cells are found in 4 to 6 nearby lymph nodes.
   N2b: Cancer cells are found in 7 or more nearby lymph nodes.
M0: No distant spread is seen.
   M1a: The cancer has spread to 1 distant organ or set of distant lymph nodes.
   M1b: The cancer has spread to more than 1 distant organ or set of distant lymph nodes, or it has spread to distant parts of the peritoneum (the lining of the abdominal cavity).

Combining the information of each letter, in a process called stage grouping, the stage is expressed in Roman numerals from stage I (the least advanced) to stage IV (the most advanced). Some stages are subdivided in letters (Table 1).

Another factor used to analyze the survival is the grade of the cancer [7]. Grade is a description of how closely the cancer looks like normal colorectal tissue when seen under a microscope. The scale used for grading a CRC goes from G1 (where the cancer looks like normal colorectal tissue) to G4 (where the cancer looks very abnormal). The grades G2 and G3 fall somewhere in between. The grade is often simplified as “low grade” (G1 or G2) or “high grade” (G3 or G4) [2]. Low-grade cancers tend to grow and spread more slowly than high-grade cancers. 

### 1.1. Classical Model of Carcinogenesis: Multihit Hypothesis

**Figure 1:**
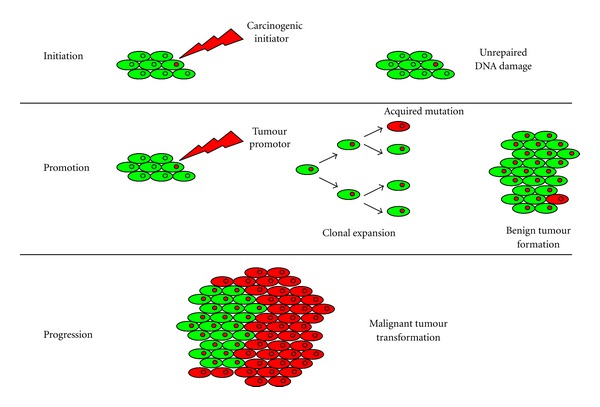
Classical theory of carcinogenesis. Cancer is generated by a three-step process: initiation, promotion, and progression. Several mutations are needed to develop a cancer.

Cancer is classically generated by a three step process, consisting of initiation, promotion, and progression (Figure 1). A simple mutation is not enough to develop a cancer, and thus the multiple-hit hypothesis indicates that cancer is the result of accumulated mutations to a cell's DNA. This hypothesis was first proposed by Nordling [8] and later by Knudson [9].

Initiation includes the formation of a malignant cell after a carcinogenic initiator damages DNA. Carcinogenic initiators include UV light, ionization radiation, thermal disruption, or chemical sources [10]. Genotoxic initiators mutate cellular DNA by five main types of DNA damage including

- oxidation of bases (e.g., 8-oxo-7,8-dihydroguanine (8-oxoG)) and generation of DNA strand interruptions (usually produced by reactive oxygen species),
- alkylation of bases (specially methylation, e.g., in 1-methyladenine, or 7-methylguanine),
- hydrolysis of bases (e.g., deamination, depurination, and depyrimidination),
- bulky adduct formation (e.g., aristolactam I-dA adduct, or benzo[a]pyrene diol epoxy-dG adduct),
- mismatch of bases, due to errors in DNA replication.

If DNA damage remains unrepaired, a promoter induces a clonal expansion of the cell and a generation of cells with mutated genes is formed in the so-called promotion phase. Once a tumour is established, then mutation, genetic instability, or epigenetic changes can lead to new clones that contribute to tumour expansion (clonal evolution model). It should be noted that three types of genes can be altered to yield cancer.

**(1) Protooncogenes**

These are normal genes that can become oncogenes due to a mutation [11], which changes the structure and function of a normal protein and generates an oncoprotein. Comparing with the normal protein, oncoproteins can cause an increase in the protein activity, a loss in regulation, or an increased concentration (due to an increase of protein expression, of mRNA stability, or a chromosomal abnormality). Examples of proto-oncogenes include *RAS*, *WNT*, *MYC*, *ERK*, and *TRK*.

To distinguish between proto-oncogenes intrinsic in the cell and those incorporated by viruses, oncogenes are named as c-oncogenes (or cellular oncogenes) and v-oncogenes (or viral oncogenes).

**(2) Tumours Suppressor Genes**

These genes protect the cell on the path to cancer. Nevertheless, when a tumour suppressor gene is mutated, the cell can progress to cancer. Although the multiple-hit hypothesis [8] indicates that further mutations have to be observed to progress to cancer, in some cases, a mutation of a single allele can cause increased carcinogen susceptibility [12]. Examples of tumours suppressor genes include *APC*, *TP53*, *SMAD4*, *SMAD2*, and *DCC*.

**(3) DNA Repairing Genes**

Over 130 genes are thought to be involved in DNA repair mechanisms in humans. DNA repair mechanisms include single-strand DNA damage (base excision repair (BER), nucleotide excision repair (NER), mismatch repair (MMR)) and double-strand DNA damage (nonhomologous end joining (NHEJ), microhomology-mediated end joining (MMEJ), and homologous recombination (HR)) [13]. DNA repair defects can result in inactivation of tumour suppressors or activation of oncogenes, causing cancer. The main DNA repairing genes are *MSH2*, *MSH3*, *MSH6*, *MLH1*, *BLM*, and *PMS2*.

The multihit hypothesis suggests that several successive mutations in the same cell, probably about seven in the case of human cancer, would be necessary to develop a cancer [8]. Obviously, it cannot be assumed that any seven mutations will cause cancer. Only mutations which increase the ratio between cellular divisions and cellular loss in a positive direction in the environment may be expected to have this effect.

### 1.2. Modern Aspects in Colorectal Cancer

In the last decade of cancer studies, it has been observed that when normal cells progressively evolve to a neoplastic state, they acquire six biological capabilities during the multi-hit development of tumours. In fact, tumours are more than insular masses of proliferating cells, and they have often been compared to embryonic cells. They are seen as complex tissues of multiple cell types interacting with one another. 

For CRC, cells develop an ordered series of events called “adenoma-carcinoma sequence,” which begins with the transformation of normal colonic epithelium to an adenomatous intermediate and to an adenocarcinoma. This evolution to the neoplastic state requires a genomic instability, which will be described later.

The six biological capabilities acquired by a tumour include sustaining proliferative signalling, evading growth suppressors, activating invasion and metastasis, enabling replicative immortality, inducing angiogenesis and resisting cell death [14]. Some of these capabilities are acquired due to changes in the nodes or checkpoints, which are directly related to the signalling processes. Therefore, to prevent cancer, it is very important to study these signalling processes.

**Figure 2:**
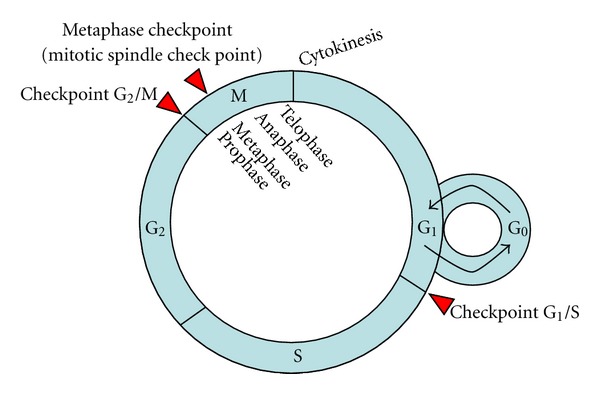
Cell cycle and its control. G_0_ stands for gap 0, G_1_ for gap 1, S for synthesis, G_2_ for gap 2, and M for mitosis. Cell cycle includes interface (G_1_, S, and G_2_), and M (including prophase, metaphase, anaphase, and telophase). Nondividing cells (G_0_ or resting phase) are not considered to be in the cell cycle. Cytokinesis follows mitosis. Checkpoint G_1_/S decides if cells go to rest (G_0_) or to S. Checkpoint G_2_/M decides if cells can begin mitosis. Checkpoint M occurs in metaphase of mitosis and it is in charge of biorientation.

Normal tissues control their ability of proliferation by the production and release of growth-promoting signals that instruct entry into and progression through the cell cycle. The cell cycle is an ordered set of events, which culminates in cell growth and division into two daughter cells. Nondividing cells (G_0_ or resting phase) are not considered to be in the cell cycle. Thus, cell cycle includes the stages G_1_-S-G_2_-M (Figure 2). G_0_, G_1_, and G_2_ stages stand for “Gap 0,” “Gap 1,” and “Gap 2” stages, where no visible changes are observed in the cell. The S stage stands for “synthesis,” and it is in this stage when DNA replication occurs. G_1_, S, and G_2_ are collectively known as interphase. The M stage stands for “mitosis,” and it is in this stage when nuclear chromosomes separate and cytoplasmic division (cytokinesis) occurs. Mitosis is further divided into 4 phases: prophase, metaphase, anaphase, and telophase. Cytokinesis is an event that directly follows mitosis, in which cytoplasm is divided into two cells.

Cell cycle is regulated by the combined action of cyclins and cyclin-dependent kinases (CDKs). Three checkpoints control the cell cycle and allow cell proliferation. G_1_ (restriction) checkpoint is located at the end of G_1_ phase, just before the entry to S phase, deciding if the cell should divide, delay division, or enter to a resting state (G_0_). G_2_ checkpoint is located at the end of G_2_ phase, triggering the start of M phase. Finally, the metaphase checkpoint (also called mitotic spindle checkpoint) occurs in the metaphase of mitosis.

A defect in the cell cycle checkpoints, a DNA damage, or a mutation nonrepaired lead to genomic instability [15]. The molecular origins of CRC are relatively well characterized and strongly related to accumulation of genetic mutations in CRC progression of three separate and distinct underlying pathways of genomic instability: chromosome instability (CIN), microsatellite instability (MSI), and CpG island methylator phenotype (CIMP) [16].

 The consequence of CIN, the prevalent phenotype in most human solid tumours (85% of CRC), is an imbalance in chromosome number (aneuploidy), subchromosomal genomic amplification, and a high frequency of loss of heterozygosity (LOH). Nevertheless, no quantitative criteria are known to define a “CIN-positive” tumour, and approaches to measure CIN include cytometry, karyotyping, loss of heterozygosity analysis, fluorescent *in situ* hybridization and genomic hybridization, which propose new subcategories of CIN-high and CIN-low for CIN-positive tumours. MSI phenotype (15% of CRC) is characterized by increased mutation rate at the nucleotide level, mainly in repetitive microsatellite sequences, and absence of big chromosomal aberrations. The consequence of MSI is instability in stretches of DNA microsatellites, which are not repaired due to a defect in the DNA mismatch repair system. CIMP consists in a gene silencing due to hypermethylation of CpG islands. Because the definitions of the three events are not exclusive, a tumour can show multiple pathways, and up to 25% of MSI colorectal cancers can show chromosomal abnormalities.

### 1.3. Chromosome Instability (CIN)

Chromosome instability (CIN) is present in the majority of all CRC (about 85%). Although more than 100 genes can cause CIN in *Saccharomyces cerevisiae*, only a limited number have been implicated in human tumours. CIN pathway is thought to be largely driven by mutational events in oncogenes and tumor suppressor genes [17].

CIN describes a dynamic state in which cells continuously gain or lose whole chromosomes, or parts of chromosomes [18]. CIN tumours contain both numerical (nCIN) and structural (sCIN) chromosome changes. Numerical CIN (nCIN) is related to gain or loss of whole chromosomes, and therefore being the chromosome number different to 2n. Therefore, some methods based on detection of chromosome number have been used to find CIN. Nevertheless, aneuploidy, an abnormal chromosomal number, is not synonymous to CIN, although CIN is the main cause of aneuploidy. Thus, CIN determination methods based on aneuploidy have to be taken with care. Molecular mechanisms for nCIN include weakening of the mitotic checkpoint, aberrant sister chromatid cohesion, centrosome amplification and improper attachment of chromosomes to the mitotic spindle [19–22].

Structural CIN (sCIN) is refereed to an increase rate of formation of structural abnormal chromosomes. A key feature associated with sCIN is the formation of “reactive” chromosomes after chromosome breaks. These “reactive” chromosomes result in breakage-fusion-bridge (BFB) cycles, which can increase the genomic rearrangements [23]. BFB cycles have been related to sCIN and associated to intratumour heterogeneity. Three molecular mechanisms have been described for sCIN: telomere dysfunction, fragile sites, and aberrant DNA repair pathways.

During mitosis, both copies of a pair of duplicated chromatids attach via their kinetochores (large protein complexes assembled onto the centromere at the site of the central constriction) to opposite poles within a microtubule-based mitotic spindle. This process, called biorientation, is essential to maintain diploidy [24]. In fact, to obtain a correct biorientation, it is essential a correct mitotic spindle checkpoint. Mitotic checkpoint complex (MCC) consists of a protein complex formed by BubR1 (product of the *BUB1B* gene), Bub3, Mad2, and Cdc20 [25–32]. Anaphase Promoting Complex/Cyclosome (APCC) polyubiquitinates several substrates (e.g., Securin and Cyclin B) to target them for destruction by the proteasome [33, 34]. This APCC complex is activated by Cdc20, but as Cdc20 is tightly bound to MCC, APCC remains inactive and Securin and Cyclin B are maintained. Securin is an inhibitor of Separase, a caspase-like protease that cleaves the Cohesion molecules which holds sister chromatids together at the centromere [35]. Cyclin B is the activator of the major mitotic kinase Cdk1 (cyclin-dependent kinase 1), and destruction of Cyclin B inactivates Cdk1, causing mitotic exit. 

BubR1 is part of the MCC, but it is thought to inhibit the APCC by blocking of substrate access. Bub1, in turn, participates in inhibition of the APCC by phosphorylating and inactivating Cdc20 [36]. Bub3 recruits both BubR1 and Bub1 to unattached kinetochores [37]. Several other proteins have been proposed to participate in this checkpoint signalling. One of these is Aurora B, a protein kinase that has an important role in regulating kinetochore-microtubule interactions in higher eukaryotes. Another protein, the retinoblastoma (RB) tumour suppressor, has been also related to CIN. RB tumour suppressor is the downstream mediator of a cellular pathway that is thought to prevent cancer by controlling the ability of cells to enter or exit the cell cycle in G_0_/G_1_ [38]. Recently, there are accumulating evidences suggesting that RB, its family members' p107 and p130, and their partners, the E2F family of transcription factors, may have important cellular functions beyond the G_1_/S transition of the cell cycle, including during DNA replication and at the transition into mitosis.

CIN had been examined by several techniques [39, 40], including karyotyping [41], DNA content flow cytometry (FCM) [42], interphase fluorescence *in situ* hybridization (FISH) [3, 4, 43, 44], and gain/loss of DNA by comparative genome hybridization (CGH) [11, 45], which allow detection of both numerical and structural chromosome aberrations in these preneoplastic lesions [18]. In particular, studies for CRC brought evidences of gains of chromosomes 7, 13, and 20, loss of chromosome 18, and deletion of 1p (see also The Mitelman Database of Chromosome Aberrations in Cancer at http://cgap.nci.nih.gov/Chromosomes/Mitelman) [46].

The CIN molecular mechanism has been predicted to be associated to cell cycle mitotic checkpoint gene mutations, to telomere dysfunctions, microtubule dynamic instability, kinetochore structure and function, chromosome condensation and sister-chromatid cohesion [24, 47]. Among the genes reported to monitor genome integrity and CIN, *TP53* inactivation, in association with the dysfunction of telomeres, has been suggested as one of the most important driving forces. Nevertheless, inactivation of *TP53* is rare in colorectal adenomas [42, 48, 49]. In addition to the investigation of mitotic genes, which were found to be rarely mutated [21, 50], genes in the other cell cycle phases, as for example *RB1* [51], *MYC* [52], *CCDN1* [53], and *CCNE1* [54], have been investigated and proposed to lead to CIN by uncoupling cell cycle progression and mitotic control.

The mutations of *KRAS* and *APC* have been also proposed to have a role in CIN in CRC [4, 11, 18–20, 22–44, 55, 56].

### 1.4. Microsatellites Instabilities (MSIs)

The MSI pathway is not as common as CIN in CRC (about 15%), but it is an extremely useful screening tool for the detection of families affected by hereditary nonpolyposis CRC (HNPCC), or Lynch syndrome, due to a defect in DNA mismatch repair (MMR) system. MSIs are insertion and deletion mutations at microsatellites, because these structures are particularly prone to DNA replication. Microsatellites are defined as stretches of DNA sequence where a single nucleotide (mononucleotides) or units of two or more nucleotides (di-, tri-, tetra-, pentanucleotides, etc.) are repeated in the genome. Repeated units with as many as several hundreds have been also classified as microsatellites, which indicate that the microsatellite length can be very variable [57].

There are at least 500,000 microsatellites in the human genome, either in intergenic or noncoding regions (with unknown functional significance) as well as in gene-encoding regions (coding microsatellites, cMS). They are commonly located in the introns of genes, but there are numerous examples of microsatellites in promoters, untranslated terminal regions, and in the coding exons themselves. Insertions or deletions in cMS result in the production of a truncated and therefore inactive protein. Examples of genes containing coding repeats that are targets for mutation in CRC with MSI include genes related with DNA repair (*RAD50*, *MSH2*, *MSH3*, *MSH6*, *MLH1*, *BLM*, *PMS2*), apoptosis (*APAF1*, *BAX*, *BCL10*, *Caspase-5*), signal transduction (*TGF*β*RII*, *ACTRII*, *IGFIIR*, *WISP-3*), cell cycle (*PTEN*, *RIZ*), and transcription factors (*TCF-4*) [58].

A standard test for MSI is the proposed at the National Cancer Institute (NCI) in 1997, also known as the Bethesda panel. This is a panel consisting of two mononucleotide repeats (*BAT25*, *BAT26*) and three dinucleotide repeats (*D2S123*, *D5S346*, *D17S250*) [59]. By using this panel, instability can be classified in high-level MSI (MSI-H) with instability at the five Bethesda panel, and low-level MSI (MSI-L) with instability at only one of the five Bethesda panel. Microsatellite stable (MSS) presented none positive marker in the Bethesda panel [60]. While almost all MSI-H tumours are MMR deficient, most of all MSI-L tumours have no MMR defect. The use of *BAT26* alone is not recommended for diagnostic MSI screening because of polymorphism in approximately 10 percent of African population that can lead to false positives for MSI.

For this reason, Suraweera et al. [61] proposed a new five-marker or pentaplex panel for MSI screening that comprises the mononucleotide repeats *BAT25*, *BAT26*, *NR21*, *NR22,* and *NR24*. The pentaplex assay is commercially available and has been used for several years by routine in anatomical pathology laboratories, although the simultaneous assessment of two markers (*BAT26* and *NR24*) has been shown to be as effective as the pentaplex panel for diagnosis of MSI.

The MSI-H phenotype is observed in about 15% of sporadic CRC and in all CRC developing in individuals with the inherited cancer-predisposing Lynch syndrome [62].

### 1.5. CpG Island Methylator Phenotype (CIMP)

CIMP consists in a gene silencing due to hypermethylation of CpG islands [63]. CpG islands are regions of nucleic acid that are often located proximally to the transcription start site of genes that contain a high frequency of CG dinucleotides. Although the molecular determinants of CIMP in tumour cells are only beginning to be elucidated, the best understood component is the transcriptional repression of a growing list of tumour suppressor and candidate tumour-suppressor genes. This suppression is associated with abnormal methylation of nucleic acid at certain cytosine residues of the cytosine and guanine-rich regions called CpG islands, often found in the promoter regions of these genes [64].

In most mammalian genes, these CpG regions are normally kept free of methylation, or an epigenetic mechanism may repress gene transcription in normal cellular processes. Nevertheless, in cancer cells, CpG islands in various tumour-suppressor genes are frequently densely methylated, which results in repression of transcription. By this mechanism of “silencing,” the expression of these tumour-suppressor genes in the cancer cell can be reduced or eliminated.

The identification of genes that are specifically hypermethylated (which results in gene silencing) or hypomethylated (which results in increased transcription) might lead to the discovery of new factors that are important for tumour initiation and progression [65]. Of particular importance is the identification of genes, the silencing of which confers a survival benefit to the cells, contributing to a neoplastic phenotype and facilitating tumour progression by allowing the accumulation of additional genetic and/or epigenetic hits. Genome methylation patterns are also being developed as biomarkers for tumour type, as markers for risk assessment, early detection and monitoring of prognosis, and as indicators of susceptibility or response to therapy [66].

MicroRNAs have been shown to act as oncogenes or tumour suppressing genes in cancer. The number of microRNAs (miRNAs) with putative growth-inhibitory functions undergoing promoter CpG island hypermethylation in human cancer is growing fast and more detailed biological studies are necessary. The recognition of miR-155 with oncogenic function has been demonstrated through their specific downregulation of MLH1, MLH2, and MSH6, core components of DNA mismatch repair, and it has been implicated in the pathogenesis of nonpolyposis colorectal cancer (HNPCC). miR-373 has also been suggested to have oncogenic activity in several types of cancers, including colon cancer [67, 68]. The miR-34 family of miRNAs is another group of miRNAs that function as important tumor suppressors in many types of cancers [69]. The tumor suppressor function of this family of miRNAs is also mediated through their down-regulation of multiple targets in the apoptosis and cell cycle control pathways, including Bcl-2, cyclin D1, cyclin E2, CDK4, CDK6, c-MYC, E2F3, MET, MYCN, Notch, and SIRT1 [69–71].

## 2. Types of Colorectal Cancer

Most cases (about 95%) of CRC are sporadical (with no background of a family history of the disease). In these cases, mutated genes occur by chance (somatic mutation). Familiar CRCs are less common (about 5%) and occur when gene mutations are passed within a family from one generation to the other. In these cases, mutated genes (germline mutation) are inherited. Inherited CRCs include two hereditary nonpolyposis colorectal cancer (HNPCC) (or Lynch syndromes I and II), familiar adenomatous polyposis (FAP), MYH-associated polyposis (MAP), Peutz-Jeghers syndrome (PJS), and juvenile polyposis syndrome (JPS).

### 2.1. Sporadic CRC

Around 60% to 80% of MMR-deficient tumours are caused by somatic events affecting both alleles, and therefore not inherited. The overwhelming majority of these are due to hypermethylation of *MLH1*. A simple way to test methylation is the methylation-specific PCR method (MSP) [76]. To distinguish between the hereditary (Lynch syndrome) and the sporadic form, other methylations are analyzed by immunohistochemistry, including the *BRAF* gene (the common somatic V600E mutation), which is present in 40–60% of MSI positive tumours and in 69% of tumours with absence of MLH1, but never in Lynch syndrome [77]. Losses or gains of defined chromosomal regions, or loss of heterozygosity (LOH), have been also observed in human sporadic colorectal adenomas of very small size [48, 78].

### 2.2. Hereditary Nonpolyposis Colorectal Cancer (HNPCC): Lynch Syndromes

Henry Lynch described in 1966 a familiar colon, endometrial and gastric cancer without colonic polyposis. These cancer family syndromes were later named Lynch syndromes 1 and 2, and also designed as HNPCC (hereditary nonpolyposis colon cancer). Over 90% of all colorectal cancers in HNPCC patients demonstrate MSI-H. This means that at least five genes have been mutated in HNPCC families or atypical HNPCC families. These mutations increase the risk of CRC, as well as cancers of the stomach, small intestine, liver, bile duct, urinary tract, brain and central nervous system, and breast.

HNPCC is the most common form of hereditary colorectal cancer, and it accounts for 3–5% of all colorectal malignancies. It is inherited as an autosomal dominant disease, as a result of defective mismatch repair (MMR) caused by the failure of one of the four main MMR genes (*MSH2* on chromosome 2p16, *MLH1* on chromosome 3p21, *MSH6* on chromosome 2p16, or *PMS2* on chromosome 7p22) [79].

Most mutations that cause Lynch syndrome are found in the *MLH1* or *MSH2* genes, but not all families that appear to have Lynch syndrome will have mutations in *MLH1*, *MSH2*, *MSH6*, or *PMS2*. Research is ongoing to identify other genes associated with Lynch syndrome. From a study with patients belonging to families diagnosed with Lynch syndrome, more than 80% of tumours display MSI [80]. The most common cause of the absence of MSI in Lynch syndrome is a false negative, resulting from either an inadequate number of microsatellite markers or an inadequate proportion of tumour cells in the sample. A less common cause is that the tumour is a phenocopy, that is, is a spontaneous (sporadic) tumour in an individual with Lynch syndrome. In practice, unless there is a strong family history of Lynch syndrome-associated cancer, patients with the positive five-marker Bethesda panel or another panel of high quality do not need further evaluation for a possible diagnosis of Lynch syndrome.

Patients with MSI-L can also be diagnosed with Lynch syndrome if they show mutations in *MLH1* or *MSH2* (Muir-Torre syndrome) or in *MSH6* or *PMS2* (Turcot syndrome) [81]. Muir-Torre syndrome is a type of HNPCC, mainly characterized by mutations in genes *MLH1* or *MSH2*, although some cases have been described with mutations in *MSH6* [82]. Muir-Torre syndrome patients have also risk for developing certain skin changes in adulthood in the sebaceous glands, which include sebaceous adenomas, sebaceous epitheliomas, sebaceous carcinomas, and keratoacanthomas. Turcot syndrome is a type of both HNPCC and FAP characterized by multiple adenomatous colon polyps, and increased risk of CRC and brain cancer [83]. The type of brain cancer depends on whether the Turcot syndrome is more similar to HNPCC (glioblastoma) or FAP (medulloblastoma). In families with glioblastoma and other features of HNPCC, mutations have been found in two genes: *MLH1* and *PMS2*. In families with medulloblastoma and other features of FAP, mutations have been mainly found in the *APC* gene.

### 2.3. Familiar Adenomatous Polyposis (FAP)

Classical familiar adenomatous polyposis (called FAP or classic FAP) was the first polyposis syndrome recognized and the best characterised. FAP is an autosomal dominant disorder caused by a mutation in the *APC* gene [84], located on chromosome 5q21. *APC* is a tumour suppressor gene, and besides being the cause of FAP, it is also involved in the early initiation of sporadic CRC. By routine screening methods, no *APC* mutation has been detected in 20–30% of classical FAP patients. However, on monoallelic mutation analysis more than 95% of FAP patients show an identifiable mutation.

The hallmark of FAP is the development of more than 100 adenomatous polyps in the colon and rectum, usually starting in the adolescence [85, 86]. Upper gastrointestinal polyps are present in nearly 90% of FAP patients by the age of 70 years. Only a small fraction of CRC is caused by FAP (<1%) and this fraction is decreasing with improved diagnostics and treatment.

There are three subtypes of classic FAP called attenuated FAP (AFAP), Gardner syndrome, and Turcot syndrome [87]. In AFAP adenomatous polyps in colon are less than 100, with 30 being average, and these polyps are developed later in life than in individual with classic FAP, although polyps may be developed as early as the late teens. A mutation in *APC* gene is also observed in AFAP. Gardner syndrome is a type of FAP also associated with osteomas (bony tumors) of the jaw, extra teeth, and soft tissue tumors including lipomas (fatty tissue) and fibromas (fibrous tissue).

### 2.4. MYH-Associated Polyposis (MAP)

MAP is a hereditary condition, caused by a specific genetic mutation of *MYH* (also called *MUTYH*, mut Y homolog), which is associated with multiple adenomatous polyps that increase the risk of CRC. The disease appears at relatively young age (between 20 and 50 years old) [88, 89]. MAP follows an autosomal recessive inheritance pattern, and to develop the disease it is needed a mutation in both copies of the gene. Thus, carriers (persons with only one copy of the gene mutation) do not develop the disease. There are two common mutations in *MYH* called *Y165C* and *G382D*.

### 2.5. Peutz-Jeghers Syndrome (PJS)

PJS is an inherited condition, caused by a specific genetic mutation of *STK11* (serine/threonine kinase 11). It is a very rare autosomal recessive disease, as it is estimated that one in 100,000 people will develop the disease. This syndrome is associated with development of hamartomatous polyps (noncancerous tumours) in the digestive tract that can later develop digestive tract, breast, or colorectal cancers. PJS tend to develop dark blue or dark brown freckling, especially around the mouth and on the lips, fingers, or toes [90, 91]. Freckles generally appear in childhood and may fade with age.

### 2.6. Juvenile Polyposis Syndrome (JPS)

JPS is a rare autosomal dominant condition characterised by hamartomatous polyps, usually within the colon but occasionally arising in the stomach, small intestine, and pancreas. These polyps are typified by a predominant stroma, cystic spaces, and an abundant lamina propria lacking smooth muscle, so distinguishing them from Peutz-Jeghers polyps. Unlike solitary juvenile polyps, which may affect up to 2% of children and adolescents and have little or no malignant potential, JPS patients have an increased risk of gastrointestinal malignancy. A mutation in either the *BMPRIA* (bone morphogenetic protein receptor, type IA) gene or the *SMAD4* gene makes more likely to develop juvenile polyps and cancer of the digestive tract [92]. Nevertheless, other genes are being studied regarding their link to JPS.

Juvenile polyps also occur as a manifestation of the dominantly transmitted familial cancer syndromes: Cowden syndrome (CS) characterised by multiple hamartomas, macrocephaly, trichilemmomas, and a high risk of benign and malignant neoplasms of the thyroid, breast, uterus, and skin [93]; Bannayan-Ruvalcaba-Riley syndrome (Bannayan-Zonana syndrome, BRRS, BZS) characterised by mental retardation, macrocephaly, lipomatosis, haemangiomas and genital pigmentation [94, 95]; and Gorlin syndrome (GS) characterised by multiple naevoid basal carcinomas, skeletal abnormalities, and odontogenic keratinocytes, macrocephaly, intracranial calcification, and craniofacial abnormalities [96, 97]. Compared with JPS the risk of gastrointestinal malignancy in CS, BRRS, and GS appears to be low. Approximately 85% of patients diagnosed with CS and 60% of the patients with BRRS have a mutation of *PTEN* (phosphatase and tensin homolog) gene. PTEN functions as a dual-specific phosphatase that removes phosphates groups from tyrosine, serine, and threonine. 

## 3. Signalling Modified by CRC Oncogenes

In the different types of CRC described above, it has been described different mutations in several genes. These mutations affected basically three signalling pathways, including the Wnt-*β*-catenin pathway (genes related: *APC*, *Axin 2*, *CTNNB1*), tyrosine kinase receptors (genes related: *K-Ras*, *ABL*, *LCK*, *SRC*, *AKT*, *RAF1*, *MOS*, *PIM1*), and TGF*β* (genes related: *TGF*β*RII*, *SMAD2*, *SMAD4*).

Other genes related with CRC include DNA mismatch repair (genes related: *MLH1*, *MLH3*, *MSH2*, *MSH6*, *PMS1*, *PMS2*) and genes related with cell cycle checkpoints and apoptotic pathways (*BAX*). Some controversial studies indicated that genes related with Hedgehog pathway might also affect in CRC. In the following, those pathways will be more detailed.

### 3.1. Wnt-*β*-Catenin Pathway

The origin of the name Wnt comes from wingless in *Drosophila melanogaster*, which is the best characterized *Wnt* gene. Aberrant activation of the Wnt/*β*-catenin signalling pathway is a necessary initiating event in the genesis of most CRC. Genetic mutations of the Wnt/*β*-catenin signalling intracellular components *APC*, *CTNNB1* (*β*-catenin encoding gene), and *Axin2* are major contributing factors for colorectal cancers [72]. Loss of function of adenomatous polyposis coli (*APC*) is responsible for FAP and 90% sporadic colorectal cancer.

**Figure 3:**
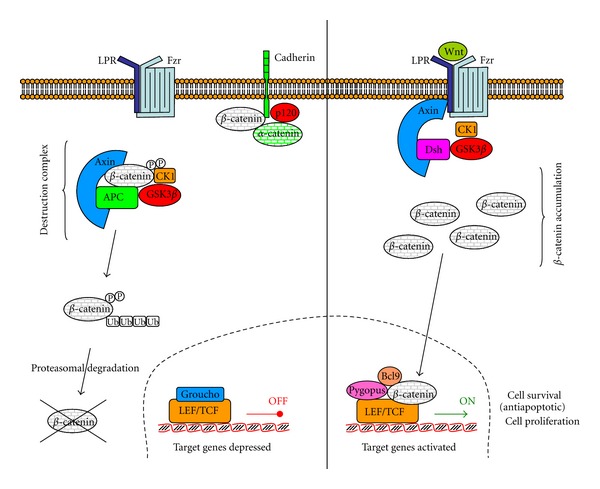
Canonical Wnt-*β*-catenin pathway. Wnt signaling pathway is shown in the “OFF” (left hand side) and “ON” (right hand side) states. In the absence of a Wnt signal, the destruction complex phosphorylates and ubiquinates *β*-catenin, being therefore destroyed by the proteasome. In the presence of a Wnt signal, as the dishevelled protein (Dsh) recruits the Axin2 and inhibits GSK-3, *β*-catenin is not phosphorylated and therefore not destroyed. It can translocate to the nucleus and activate transcriptions genes (adapted from [72]).

 Wnts are powerful regulators of cell proliferation and differentiation, and their canonical signalling pathway (Figure 3) involves proteins that directly participate in gene transcription. The main player is *β*-catenin, which is a transcription factor accumulated after 2 h of Wnt incubation [98]. Nineteen *Wnt* genes exist in mammalian genomes, and the diversity of their functions is exemplified by mutations that lead to several developmental abnormalities [99]. Wnts are secreted proteins, palmitoylated on a cysteine [100]. The lipid is important in their activity, as an enzymatic removal of the palmitate or a site-directed and natural mutation of the cysteine results in a loss of Wnt activity. Signalling is initiated by Wnt ligand binding to two receptor molecules: Frizzled proteins and low density lipoprotein receptor-related proteins 5 or 6 (LRP5 or LRP6).

Frizzled proteins (Fzd receptors) are a family of G-protein-coupled receptors, with seven transmembrane domains. All Frizzled proteins share a conserved region of 120 amino acids in the extracellular domain, with a motif of 10 invariantly spaced cysteines (called the cysteine-rich domain, CRD) [101]. The CRD domain is necessary and sufficient for Wnt ligand binding [102]. The ten members of the Fzd receptors interact with the nineteen Wnt to activate canonical and/or noncanonical Wnt signalling. Fzd7 plays an important role in colorectal cancer development and metastasis. The Fzd7 protein is abundantly expressed in colon cancer tissues and various colon cancer cell lines that also contain mutated *APC* or *CTNNB1*. Herbergs et al. [44] examined the mRNA levels of Fzd7 in 135 primary colorectal cancers by real-time PCR and found that the Fzd7 mRNA levels were significantly higher in stage II, III, or IV tumors than in nontumor tissues and that overall survival was shorter in those patients with higher Fzd7 expression. Fzd receptors can respond to Wnt proteins only in the presence of the low density lipoprotein receptor related proteins 5 or 6 (LRP5 or LRP6) to activate the canonical *β*-catenin pathway [103, 104].

The main point of the Wnt pathway is that in absence of Wnt *β*-catenin is sequestered in a “destruction complex” that contains APC (adematous polyposis coli), adenomatous polyposis coli tumour suppressor (Axin2), glycogen synthase kinase-3*β* (GSK3*β*), and casein kinase 1 (CK1), also formed when Wnt proteins are unable to bind to their receptors. The formation of this “destruction complex” induces the phosphorylation of *β*-catenin by CK1 and GSK3*β*, in particular at serine-675 [75]. Phosphorylated *β*-catenin is recognised by *β*-TrCP (*β*-transducin repeats-containing protein), an F-box component of the E3 ubiquitin ligase complex, which promotes *β*-catenin ubiquination and degradation by the ubiquitin-proteasome system [43].

The binding of Wnt to receptors to form the ternary complex (Wnt-Fzd-LRP5/6) leads to downstream evasion by *β*-catenin from degradation in the cytoplasm [105]. The adaptor protein dishevelled (Dsh) is activated and recruits Axin2, which forms a complex with Dsh [106]. In this complex, Dsh is activated and GSK3*β* is inhibited. These events reduce *β*-catenin phosphorylation and its consequent degradation. Mammalians contain three dishelled proteins, named Dsh-1, Dsh-2, and Dsh-3. Sirtuin 1 (SIRT1) is a NAD^+^-dependent histone deacetylase that regulates Dsh and Wnt signalling [107]. SIRT1 is associated with microsatellite instability and CpG island methylator phenotype in human colorectal cancer [108].

Despite the Wnt canonical pathway, there are many other Wnt noncanonical pathways, but the two best-studied pathways are the planar cell polarity (PCP) (or Wnt/JNK) and the Wnt/Calcium pathway [109, 110]. Identified in colon carcinoma cells and named colon carcinoma kinase-4, PTK7 (protein kinase 7) has recently been analyzed as a Wnt coreceptor in the non-canonical PCP [111]. This pathway is related to JNK (c-Jun N-terminal kinase) that belongs to the mitogen-activated protein kinase (MAPK) family.

### 3.2. Tyrosine Kinase Receptors Pathway

The receptors for many polypeptide growth factors and hormones are proteins with a single transmembrane domain and an intrinsic tyrosine kinase activity. Those receptors include epidermal growth factor receptor (EGFR), vascular endothelial growth factor receptor (VEGFR), platelet-derived growth factor receptor (PDGFR), and fibroblast growth factor receptor (FGFR). Insulin-like growth factor receptor (IGFR), a dimeric receptor, is also another tyrosine kinase receptor. Human epidermal growth factor receptor (HER) is one member of a family of four related proteins, termed the ErbB/HER receptors (because of their similarity to the *v-ErbB* oncogene of avian erythroblastosis virus that induces erythroid leukemia in birds). The link of ErbB2/HER2 with cancer is also observed in human, as overexpression of the human ErbB2 gene, which encodes the human EGFR (also known as HER2), is related with cancer [112].

**Figure 4:**
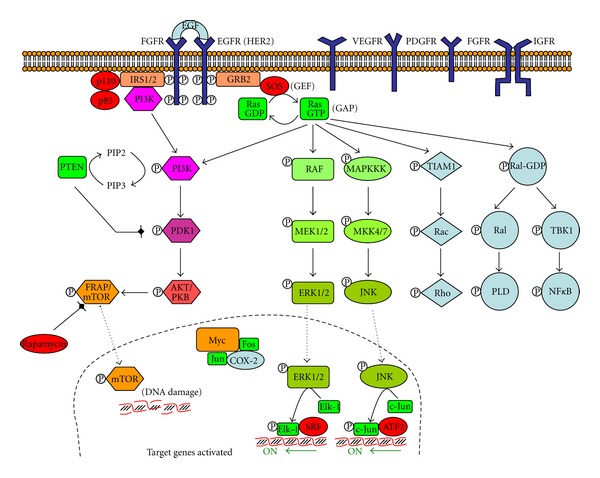
Tyrosine kinase receptors pathway. Tyrosine kinase receptors include the following dimmers receptors: EGFR, epidermal growth factor receptor (also known as HER2 or ErbB2); HER, human epidermal growth factor receptor; VEGFR, vascular endothelial growth factor receptor; FGFR, fibroblast growth factor receptor; PDGFR, platelet-derived growth factor receptor. IGFR, insulin growth factor receptor, is also a tyrosine kinase receptor. When a growth factor binds to the receptor, a cascade of phosphorylations is initiated, which finishes with activation of transcription genes (adapted from [73]).

When a growth factor binds to the extracellular domain of tyrosine kinase receptor, it triggers dimerization with another tyrosine kinase receptor, which phosphorylates the neighbour receptor (autophosphorylation) on several tyrosine residues (Figure 4). Cytoplasmic proteins of the growth factor signalling pathway typically contain similar domains as the protein SRC (pronounced “sarc,” as it is the short for “sarcoma”). These domains are called SH2 (SRC homology 2 domain), which binds to phosphorylated tyrosine, and SH3 (SCR homology 3 domain), which binds to a region in a protein that has polyproline helix secondary structure. GRB2 is a protein that contains SH2 and SH3 domains and can form a bridge between the receptor and a guanine exchange factor (GEF), which is able to exchange GDP for GTP in a GTP activating protein (GAP). SOS (son of sevenless) is the main Ras GTPase-activating protein (RasGAP). Thus, SOS activates Ras.

There are three different Ras genes in humans: *H-Ras*, *N-Ras*, and *K-Ras*. About 30% of all human tumours involve cells expressing mutated Ras oncogenes. When bound to GTP, Ras stimulates a family of serine/threonine protein kinases that trigger the mitogen-activated protein kinase (MAPK) cascade. This cascade includes the initial Ras-activated kinase RAF-1 [113], a mitogen-activated protein kinase kinase kinase; MEK, an intermediate mitogen-activated protein kinase kinase; and ERK, a mitogen-activated protein kinase, which phosphorylates multiple target proteins in cytosol and nucleus. In the nucleus, ERK phosphorylates Elk-1, a transcription factor, which activates several genes. Another mitogen-activated protein kinase (JNK, c-Jun N-terminal kinase) phosphorylates c-Jun in the nucleus, another transcription factor. Despite the RAF-MEK-ERK and the MAPKKK-MKK-JNK, Ras can also activate the PI3 K-PDK-AKT-mTOR cascade, the TIAM1-Rac-Rho cascade, and the Ral-PLD, the TIAM-Rac-Rho, and the TBK1-NF*κ*B cascades [114]. 

PI3 K-PDK-AKT-mTOR is a downstream target of EGFR, activated in cancer. Phosphatidylinositol 3-kinase (PI3 K) phosphorylates PIP2 to PIP3. The tumor suppressor gene PTEN (phosphate and tensin homologue) antagonizes the PI3 K/AKT signalling pathway by dephosphorylating PIP3 to inhibit activation of AKT with hyperactivation of PI3K signalling. The final product, mTOR (mammalian target of rapamycin), produces DNA damage [115, 116].

Tyrosine kinase receptors trigger the pathway in presence of the growth factor, but mutated tyrosine kinase receptor genes can result in oncogenes, which may express truncated receptors with tyrosine kinase activity in absence of the growth factor. Normal termination of the activity includes internalization by endocytosis of the receptor and growth factor, followed by a degradation in lysosomes, which is not performed for the oncoprotein. Many oncogenes encode several members of this signal transduction, including the nonreceptor protein kinases and GTP-binding proteins [117]. The nonreceptor protein kinases are of two types: tyrosine kinases (e.g., *ABL*, *LCK*, and *SRC*) and serine and threonine kinases (e.g., *AKT*, *RAF1*, *MOS*, and *PIM1*). Proteins involved in signal transduction become oncogenic if they bear activating mutations. Important examples are PI3 K, AKT, and SGK.

### 3.3. TGF-*β* Pathway

**Figure 5:**
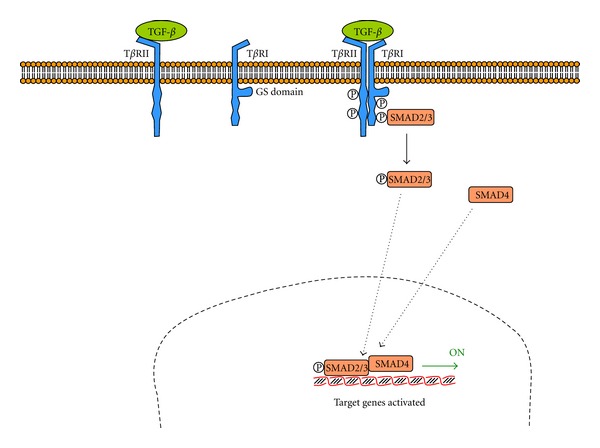
Transforming growth factor-*β* (TGF-*β*) pathway. TGF-*β* receptor is a dimer formed by T*β*RII (with a binding site for TGF-*β*) and T*β*RI (with a glycine/serine rich domain). The complete receptor is autophosphorylated and recruits and phosphorylates a gene regulatory protein called SMAD (adapted from [74]).

Transforming growth factor-*β* (TGF-*β*) has a receptor with an intrinsic serine/threonine kinase activity. Two types of receptors have been described, namely, T*β*RI (with a glycine/serine rich domain) and a T*β*RII (which can bound TGF-*β* independently). When TGF-*β* is bound to T*β*RII, a T*β*RI is recruited and the glycine/serine-rich domain is phosphorylated (Figure 5). Phosphorylated T*β*RI recruites and phosphorylates a gene regulatory protein named SMAD. In the dephosphorylated state, SMAD adopts a folded conformation and cannot bind to DNA. But when it phosphorylates, the unfold form forms dimeric complexes with other SMAD, resulting in translocation into the nucleus and interaction with other gene regulatory proteins to modulate the transcription of several genes [74].

### 3.4. Apoptotic Pathways

If proliferative signalling and evading growth suppressors are cancer capabilities related to cell cycle, whose signalling has been briefly described above, enabling replicative immortality and resisting cell death are capabilities related to apoptosis.

Apoptosis defined as a programmed cell death is characterized by many morphological changes, such as change in mitochondrial membrane potential, activation of caspases, DNA fragmentation, membrane bebbing, and formation of apoptotic bodies. Apoptosis may be activated by extrusion of cytochrome c through pores generated in the outer mitochondrial membranes by the Bcl-2-like (B-cell lymphoma 2-like) proteins Bak (Bcl-2 homologous antagonist killer) and Bad (Bcl-2 associated death promoter). There are about 24 Bcl-2-like proteins in humans, which are divided in (i) antiapoptotic proteins such as Bcl-2 and Bcl-X_L_, (ii) proapoptotic pore-forming proteins as Bax (Bcl-2 associated x protein) and Bak, and (iii) pro-apoptotic facilitator proteins, such as Bid, Bad, PUMA, and Noxa. Outside the mitochondria, cytochrome c binds to Apaf1 (apoptotic protease-activating factor 1) and procaspase 9, to form the apoptosome.

The transcription factor p53 is an important inducer of apoptosis with DNA damage. Mdm2, a E3 ligase, is an ubiquitinase pathway, which binds to p53 and promotes its polyubiquitination and degradation by proteasomes, keeping low p53 concentration. Phosphorylation of p53 by protein kinases (such as ATM, ATR, and other DNA protein kinases (DNA-PKs)) prevents the binding of Mdm2 and increases the activity of p53 [118]. Some transcription factors, such as Myc and E2F, increase the synthesis of the protein Arf, which binds to Mdm2 and prevents degradation of p53.

### 3.5. Hedgehog Pathway

**Figure 6:**
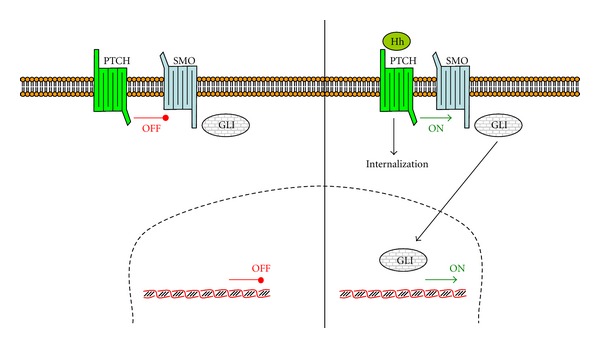
Hedgehog pathway. In absence of Hedgehog (Hh), PTCH1 binds to SMO, whereas in presence of Hh, the complex Hh-PTCH1 internalizes and releases SMO. Free SMO activates a family of transcription factors, including GLI (adapted from [75]).

Activation of Hedgehog (Hh) pathway (Figure 6) seems also implicated in colorectal cancer, although Hh and Wnt-*β*-catenin pathways rarely coexist in colorectal cancer. In fact, several studies indicate that Hh signalling components negatively regulate Wnt signalling [109]. Other studies indicate that this pathway is absent in colon, as analysis of several genes related to Hh pathway (*SHH*, *IHH*, *PTCH*, *SMO*, *GLI1*, *GLI2*, *GLI3*, *SUFU,* and *HHIP*) in diverse colon cell cultures by RT-PCR indicates that they are not present in these cells [119].

The *hedgehog* (*Hh*) gene was first identified through genetic analysis of fruit fly Drosophila, and three homologues *Hh* genes have been identified in vertebrates, named *Desert Hedgehog* (*DHH*), *Indian Hedgehog* (*IHH*), and *Sonic Hedgehog* (*SHH*). Hh signalling plays important roles in tissue morphogenesis and organ formation during gastrointestinal tract development. Deregulation of the Hh pathway has been implicated in a variety of cancers.

Hedgehog binds to Patched (PTCH1), causing internalization and degradation. In absence of Hedgehog, PTCH1 forms a complex with Smoothened (SMO). Internalization of Hh-PTCH1 releases SMO, resulting in downstream activation, including the glioma-associated oncogene (GLI) zinc finger family of transcription factors [120]. 

## 4. Treatment of Colorectal Cancer

Over the past decades, significant progress has been achieved in the treatment of CRC by advances in surgery, radiotherapy, and systemic treatment. Surgery with curative intent is the main treatment of stages I–III colon cancer, but chemicals can be also used in many cases. It is not the aim of this paper a full description of the treatment used in CRC, although some of the chemicals used will be described in the following.

Initially, adjuvant chemotherapy consisted of 5-fluorouracil (5FU), 5FU plus levamisole, or 5FU plus leucovorin. This benefit can also be achieved by capecitabine monotherapy. The combination of 5FU plus the platinum compound oxaliplatin increased the three-year disease-free survival with approximately 6%. For patients with distant metastatic CRC there are no curative treatment options and 5FU has been the standard care for decades, resulting in a survival benefit of more than 6 months. Chemotherapy with oxaliplatin and irinotecan is also very common [121].

The development of new targeted drugs, such as antibodies against vascular endothelial growth factor (VEGF) and epidermal growth factor receptor (EGFR), has added further benefits to patients with metastatic CRC. Several checkpoint kinase inhibitors or antimetabolite have entered to clinical trials and their progress has been extensively reviewed [122].

Few studies have been done analysing inhibition of cell proliferation by ribose-5-phosphate synthesis inhibition [123–126]. In fact, many treatments are based on the inhibition of the nitrogenated base synthesis, so that the nucleotides are not synthetized and they are not incorporated to DNA of the growing cancer cell. But there are few treatments regarding to the inhibition of the other part of the nucleotide: ribose-5-phosphate (or its derivate compound deoxyribose-5-phosphate). In our laboratory, we study the effect of nonoxidative and oxidative pentose phosphate pathway in cancer resistance, using dehydroepiandrosterone and oxythiamine as inhibitors [127]. These compounds could be a good complement to the treatment.

Other treatments are based on the antioxidant properties of flavanols and other natural compounds. In this sense, there are many studies related in the beneficial properties of green tea, red wine, chocolate, and other natural products [128].

### 4.1. Fluoropyrimidines

5FU belongs to the class of antimetabolite drugs and it is administered intravenously. The drug is an analogue of uracil and uses the same facilited transport mechanism for entering the cell. Uracil is incorporated into RNA and methylated to generate thymidine for DNA production [121].

Capecitabine is an oral fluoropyrimidine, and it is absorbed through the intestine as a product. By a three-step enzymatic process capecitabine is converted to 5FU. This conversion begins in the liver by the action of a carboxylesterase and cytidine deaminase to yield 5′-deoxy-5-fluorouracil. Finally, the action of thymidine phosphorylase and/or uridine phosphorylase converts this last compound to the antimetabolite 5FU. This last conversion takes place in tumours as well as in normal tissue. In terms of efficacy, this gives capecitabine a theoretical advantage compared to 5FU.

### 4.2. Platinum Compounds

Oxaliplatin ([1R,2R)-cyclohexane-1,2-diamine](ethanedioato-O,O′)platinum (II)) is a platinum compound that shows *in vitro* and *in vivo* antitumour activities in CRC, where other platinum compounds, cisplatin and carboplatin, failed to show any activity [42]. Moreover, oxaliplatin proved to be synergistic with other anticancer agents, including 5FU and irinotecan.

The main mechanism of action of oxaliplatin is mediated through the formation of DNA adducts. Once inside the cell the oxaliplatin prodrug is activated by the conversion to monochloro, dichloro, and diaquo compounds by nonenzymatic hydrolysis and displacement of the oxalate group, which leads to the formation of DNA adducts. The kinetics of hydrolysis differs amongst platinum compounds, being slower for oxaliplatin than for cisplatin. An important factor is the induction of apoptosis by the primary DNA-Pt lesions, which is possibly enhanced by a contribution of targets other than DNA.

### 4.3. Irinotecan

Irinotecan is a topoisomerase 1 (topo-I) inhibitor. It acts as a prodrug of SN-38 (7-ethyl-10-hydroxycamptothecin), which is 100- to 1000-fold more cytotoxic than the parent drug [41], and is most cytotoxic to cells in the S-phase [129]. Other topoisomerase I inhibitors include camptothecin and topotecan, which are more used for ovarian and lung cancer, than for CRC.

Irinotecan associates with the DNA-topo-I complex, and upon stabilization single stranded breaks are induced. Irreversible DNA damage occurs when DNA synthesis is ongoing and the replication fork enters a cleavable complex, resulting in double stranded breaks and ultimately cell death. Irinotecan is metabolized in blood to the active metabolite SN-38 by butyrylcholinesterases. Irinotecan and SN-38 are present in two distinguishable forms with a pH-dependent equilibrium: an active *α*-hydroxy-*δ*-lactone ring and an inactive carboxylate structure. In blood, irinotecan is predominantly present in the inactive carboxylate form, whilst SN-38 exists predominantly in the active lactone form. The lactone forms of irinotecan and SN-38 are taken up by intestinal epithelial cells and colon carcinoma cells by passive diffusion, whereas the carboxylate form is absorbed by an active pH-dependent transport mechanism.

### 4.4. Curcumin

Curcumin is currently undergoing phase II/III clinical trials in different types of cancer, including colon and cervical, with promising results in some cases [130]. The chemotherapeutic properties of curcumin have been reported as inducing lung cancer cell death through Bax upregulation and Bcl-2, Bcl-XL downregulation, causing AIF and cytochrome c release [131]. Several other intracellular targets for curcumin have also been described, including suppression of p53 and inhibition of cyclooxygenase-2 (COX-2) [130]. 

### 4.5. Statins

 “Statins,” including lovastatin, simvastatin, atorvastatin, fluvastatin, pravastatin, and cerivastatin, are inhibitors of 3-hydroxy-3-methylglutaryl-coenzyme A reductase (HMG-CoA reductase), which are widely used drugs for the treatment of lipid disorders. In the last decade, the potential of statins in the treatment of cancer has been extensively investigated [132]. The mechanism of action is related to the inhibition of HMG-CoA reductase, which inhibits the formation of farnesyl or geranylgeranyl moieties. Several signalling proteins activities are related to prenylations. Thus, the farnesylated Ras has an important function in cell growth and differentiation [133].

### 4.6. Monoclonal Antibodies against the Extracellular Domains of EGFR and VEGR

Cetuximab and panitumumab, both monoclonal antibodies that block the extracellular domain of EGFR, have been used for metastatic colon cancer in combination with fluoropyrimidines, oxaliplatin, irinotecan, or best supportive care for patients who cannot tolerate first line agents [134]. Panitumumab is a fully human monoclonal antibody specific for the extracellular domain of EGFR. Cetuximab is a mouse/human chimeric IgG1 immunoglobulin that has the added benefit of antibody-dependent cytotoxicity through activation of the host immune response [135]. Both antibodies have been effectively combined with chemotherapy in colorectal cancer [136, 137] where response has been linked to KRAS mutational status.

Bevacizumab, a humanized monoclonal antibody to VEGF, is FDA approved for use in advanced colorectal cancer [136] with any intravenous fluorouracilcontaining regimen as initial therapy. Most types of human cancer cells, including colon cancer cells, express VEGF at elevated levels, and the hypoxic state of solid tumors is an important inducer of VEGF. Bevacizumab blocks this pathway and causes modest tumor regression in metastatic colon cancer [138]. The side effects of bevacizumab, as mentioned previously, include delayed wound healing, hemorrhage, and thromboembolic events [139–141].

Several kinase inhibitors have been used to prevent one of the growth factors pathways. Sorafenib and sunitinib are multitargeted kinase inhibitors which block tumour growth and angiogenesis by inhibition of VEGFR-1, -2, and -3, and PDGF-*α* and -*β* [142]. Sorafenib inhibits also Raf and everolimus prevents the action of mTOR [73].

### 4.7. Flavanoids

Over the last decades, extensive research on plant-based medicinal compounds has revealed exciting pharmacological properties [128, 143–148]. Several flavanoids have been described as modulators of Wnt/beta-catenin signalling [149], including epigallocatechin-3-gallate (EGCG), quercitin, and baicalein. Transresveratrol (3,5,4′-trihydroxystilbene) is a polyphenol present in grape juice and red wine. Resveratrol has been shown to have beneficial effects on reduction of oxidative stress and prevention of cancer, with antioxidant and antitumorigenic properties [150].

Asiatic acid is a pentacyclic terpenoid with wide-ranging pharmacological effects such as inflammation reduction, inhibition of tumour cell proliferation, and apoptosis induction through a mitochondria-dependent pathway. Its anticancer efficacy is attributed to its ability to inhibit transcription factor NF-*κ*B, p38 MAPK and ERK kinases [151].

## 5. Conclusions

Although there are many studies related to CRC, still a lot of research has to be done in order to understand the basis of cancer. Prevention is very important in this type of cancer, being very important to perform a colonoscopy for people over 40 years old, although there are no familiar antecedents. In fact, although there are some juvenile polyposes, there is a CRC death increase over the 40, and it is better to detect the cancer in the previous stages. As there are many causes for CRC and the typology of cancer depends on the gene mutated, treatment should be also specialized, and the original causes should be known to follow one treatment or another. Surgery is the main treatment, but natural products and new chemicals can be tested, and surely that in a short future the spectatives of live for CRC patients will increase. 

## Figures and Tables

**Table 1:** Stage systems AJCC, Dukes, and Astler-Coller. AJCC system is the most used and precise staging system for CRC and combines three letters (T: for the primary tumour, N: for spread to lymph nodes, and M: for metastasis) and numbers from 0 to 4 (indicating more severity for a higher number).

AJCC/TNM		Dukes	Astler-Coller
Stage 0	Tis, N0, M0	—	—

Stage I	T1-T2, N0, M0	A	A, B1

Stage IIA	T3, N0, M0	B	B2

Stage IIB	T4a, N0, M0	B	B2

Stage IIC	T4b, N0, M0	B	B3

Stage IIIA	T1-T2, N1, M0	C	C1
	T1, N2a, M0		

Stage IIIB	T3-T4a, N1, M0	C	C1, C2
	T2-T3, N2a, M0		
	T1-T2, N2b, M0		

Stage IIIC	T4a, N2a, M0	C	C2, C3
	T3-T4, N2b, M0		
	T4b, N1-N2, M0		

Stage IV	Any T, Any N, M1a	—	D
	Any T, Any N, M1b		

## Abbreviations

AFAP: Attenuated familiar adenomatous polyposis
AJCC: American Joint Committee on Cancer
Apaf: Apoptotic protease-activating factor
APC: Adenomatous polyposis coli
APCC: Anaphase Promoting Complex/Cyclosome
Axin2: Adenomatous polyposis coli tumor suppressor
Bad: Bcl-2 associated death promoter
Bak: Bcl-2 homologous antagonist killer
Bax: Bcl-2 associated x protein
Bcl-2-like: B-cell lymphoma 2-like
BER: Base excision repair
BFB: Breakage fusion bridge
BMPR: Bone morphogenetic protein receptor
BRRS: Bannayan-Ruvalcaba-Riley syndrome (Bannayan-Zonana syndrome, BZS)
BZS: Bannayan-Zonana syndrome (Bannayan-Ruvalcaba-Riley syndrome, BRRS)
CDK: Cyclin-dependent kinase
CGH: Comparative genome hybridization
CIMP: CpG island methylator phenotype
CIN: Chromosome instability
CK1: Casein kinase 1
cMS: Coding microsatellites
COX-2: Cyclooxygenase-2
CRD: Cysteine-rich domain
CRC: Colorectal cancer
CS: Cowden syndrome
CTNNB1: *β*-catenin encoding gene
DHH: Desert Hedgehog
Dsh: Dishevelled (an adaptor protein)
EGFR: Epidermal growth factor receptor
FAP: Familiar adenomatous polyposis
FCM: Flow cytometry
FGFR: Fibroblast growth factor receptor
Fzd receptor: Frizzled receptor
GAP: GTP activating protein
GEF: Guanine exchange factor
GS: Gorlin syndrome
GSK3*β*: Glycogen synthase kinase-3*β*
HER: Human epidermal growth factor receptor
Hh: Hedgehog
HNPCC: Nonpolyposis colorectal cancer
HR: Homologous recombination
IGFR: Insulin-like growth factor receptor
IHH: Indian Hedgehog
JNK: c-Jun N-terminal kinase
JPS: Juvenile polyposis syndrome
LOH: Loss of heterozygosity
LRP: Low density lipoprotein receptor-related protein
NCI: National Cancer Institute
nCIN: Numerical chromosome instability
NER: Nucleotide excision repair
NHEJ: Nonhomologous end joining
MAP: MYH-associated polyposis
MAPK: Mitogen-activated protein kinase
MCC: Mitotic checkpoint complex
miRNA: MicroRNA
MMEJ: Microhomology-mediated end joining
MMR: Mismatch repair
MSI: Microsatellite instability
MSI-H: High-level microsatellite instability
MSI-L: Low-level microsatellite instability
MSP: Methylation-specific PCR method
MSS: Microsatellite stable
mTOR: Mammalian target of rapamycin
PCP: Planar cell polarity (or Wnt/JNK pathway)
PDGFR: Platelet-derived growth factor receptor
PJS: Peutz-Jeghers syndrome
PTEN: Phosphatase and tensin homolog
PTK: Protein kinase
RasGAP: Ras GTPase-activating protein
RB: Retinoblastoma
sCIN: Structural chromosome instability
SH2: SRC homology 2 domain
SH3: SCR homology 3 domain
SHH: Sonic Hedgehog
SIRT1: Sirtuin 1
SOS: Son of sevenless
STK: Serine/threonine kinase
topo-I: Topoisomerase 1
TGF-*β*: Transforming growth factor-*β*
*β*-TrCP: *β*-transducin repeats-containing protein
VEGFR: Vascular endothelial growth factor receptor.
